# Unspecified verticality of Franck–Condon transitions, absorption and emission spectra of cyanine dyes, and a classically inspired approximation

**DOI:** 10.1039/d0ra06774a

**Published:** 2020-11-26

**Authors:** Joseph D. Alia, Joseph A. Flack

**Affiliations:** Division of Science and Mathematics, University of Minnesota Morris 600 E 4th St USA aliaj@morris.umn.edu +1-320-589-6345; NYITCOM at Arkansas State University P. O. Box 2206, State University AR 72467 USA

## Abstract

The computed vertical energy, *E*_v,a/f_, from the equilibrium geometry of the initial electronic state is frequently considered as representative of the experimental excitation/emission energy, *E*_abs/fl_ = *hc*/*λ*_max_. Application of the quantum mechanical version of the Franck–Condon principle does not involve precise specification of nuclear positions before, after, or during an electronic transition. Moreover, the duration of an electronic transition is not experimentally accessible in spectra with resolved vibrational structure. It is shown that computed vibronic spectra based on TDDFT methods and application of quantum mechanical FC analysis predict *E*_abs_ = *hc*/*λ*_max_ with a 10-fold improvement in accuracy compared to *E*_v,a_ for nine cyanine dyes. It is argued that part of the reason for accuracy when this FC analysis is compared to experiment as opposed to *E*_v,a/f_ is the unspecified verticality of transitions in the context of the quantum version of the FC principle. Classical FC transitions that preserve nuclear kinetic energy before and after an electronic transition were previously found to occur at a weighted average of final and initial electronic state molecular geometries known as the r-centroid. Inspired by this approach a qualitative method using computed vertical and adiabatic energies and the harmonic approximation is developed and applied yielding a 5-fold improvement in accuracy compared to *E*_v,a_. This improvement results from the dominance of low frequency vibronic transitions in the cyanine dye major band. The model gives insight into the nature of the redshift when qPCR dye EvaGreen is complexed to λDNA and is applicable to the low frequency band of similar non cyanine dyes such as curcumin. It is found that the computed vibronic cyanine dye spectra from time-dependent FC analysis at 0 K and 298 K show decreased intensity at higher temperature suggestive of increased intensity with restricted motion shown when cyanine dyes are used in biomedical imaging. A 2-layer ONIOM model of the DNA minor groove indicates restricted motion of the TC-1 dye excited state in this setting indicative of enhanced fluorescence.

## Abstract 2

Insight into cyanine dye *λ*_max_ from quantum and classical FC principle; high accuracy with classically inspired approach.

## Graphical abstract

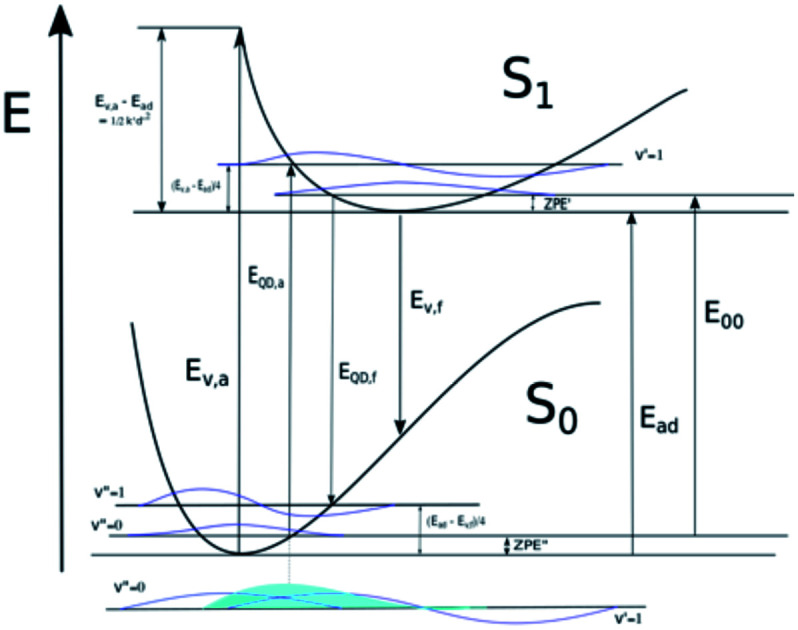

## Introduction

How should one think of the motion of nuclei during an electronic transition of a molecule? Usual statements of the Franck–Condon principle tell us change in position of nuclei is negligible during an electronic transition. Quantum and classical versions of the FC principle differ on this and whether there is even a meaningful answer. We propose that examination of this question in the context of computed vibronic absorption and fluorescence spectra of cyanine dyes helps to reveal a source of systematic error in comparison of TDDFT vertical energies to experimental spectra. We develop a qualitative classically inspired approach to a vertical energy that gives closer agreement to experiment for nine cyanine dye examples than do vertical TDDFT energies calculated from the optimized geometry of the initial electronic state. We find our applications both of the quantum mechanical FC principle and of our newly developed classically inspired approach yield high accuracy compared to experiment for the dyes studied.

### Unspecified verticality in the quantum FC principle

Franck in 1926 showed how different possible ground and excited state bond potential energy curves are the basis for understanding variation in band structure of diatomic molecules of different composition.^1^ Condon's 1926 paper builds on Franck's work showing favorable vibronic transitions to be those for which classically specified positions and momenta of nuclei have negligible change during the electronic transition.^2^ These two papers from 1926 comprise the classical Franck–Condon principle. Condon was already aware in 1926 that this treatment was provisional and would be superseded by an approach based on “the newer kinematics of Heisenberg, Born, and Jordan”. In 1928, Condon explains the “inexactness” of his previous approach and develops what is now known as the quantum mechanical Franck–Condon principle which avoids “violation of the Heisenberg indeterminacy principle” by not requiring specification of nuclear positions during a transition but instead bases the probability of a given transition on the overlap between wavefunctions of vibrations involved.^3^ Schwartz (1973) concisely summarizes this aspect of Condon's 1928 paper, “… as Condon pointed out (3), the uncertainty principle precludes the precise specification of the nuclear position and momentum. For this reason, Condon rejected the earlier statement of the Franck–Condon principle”.^4^ Schwartz goes on to show that because of energy-lifetime uncertainty, the supposed duration of an electronic transition, “10^−15^ or even 10^−18^ s depending on the authors”, is not experimentally accessible using methods yielding vibrational structure.^4^ He concludes it is not possible to experimentally compare duration of an electronic transition with period of vibrations in the transition and such a comparison is meaningless from the point of view of energy-lifetime uncertainty.^4^ The classical concept of vertical transitions is useful under some circumstances. Noda and Zare (1982) show that an averaged molecular geometry, 〈**r**〉 = 〈*v*′|**r**|*v*′′〉/〈*v*′|*v*′′〉, known as the r-centroid, gives the position of a vertical transition that preserves the kinetic energy due to motion of the nuclei before and after an electronic transition consistent with the classical Franck–Condon principle.^5^ This approach applies where one would expect a classical approach to work well, *i.e.*, small energy spacing between vibrational energy levels. Noda and Zare give several diatomic molecule examples.^5^ The Franck–Condon principle also applies to molecules with more than two atoms.

### The Duschinsky transformation

Application of the Franck–Condon principle to molecules with more than two atoms requires common normal mode coordinates between initial and final electronic states. This is accomplished with Duschinsky transformation, **Q**′′ = **JQ**′ + **K**, describing vibrational coordinates, **Q**′′, of the initial electronic state as linear combinations of those of the final electronic state, **Q**′, through application of Duschinsky rotation matrix, **J**, and shifted by the difference between initial and final electronic state equilibrium geometries along normal modes of the initial electronic state by Duschinsky shift vector **K**. Franck–Condon factors can be computed once a common normal coordinate system has been established but before this is possible, the displacement between final and initial states and their respective potential energy surfaces must be computed. There are several ways of accomplishing these goals.

### Adiabatic and vertical approximations

Approaches that account for the displacement between electronic state geometries and their respective potential energy surfaces fall into two main categories, adiabatic and vertical.^6,7^ Adiabatic methods require optimized equilibrium geometries for both states. Vertical methods extrapolate final state displacement and vibrations from the initial state optimized geometry.

Adiabatic Hessian (AH) methods include normal modes of each electronic state according to the Hessian at their respective equilibrium geometries. For adiabatic shift (AS) methods, the two electronic states are taken to have the same PES only displaced.

Vertical Hessian (VH) methods extrapolate the displacement between electronic state geometries and the vibrational modes of the final electronic state based on the final state PES at the equilibrium geometry of the initial electronic state while vertical gradient (VG) methods make a similar extrapolation for the final state but assuming both states have the same PES but displaced.^7^

The Duschinsky shift vector is explicitly calculated if adiabatic methods are used while it is only approximated in vertical methods. Adiabatic approximations, moreover, apply the harmonic approximation where it is most valid and may therefore be expected to give more appropriate computed overlap between final and initial state wavefunctions. Vertical methods, correspond to the short-time picture of spectroscopy, and can describe higher frequency lines resolved at shorter time scales but if low frequency lines, the 0–0 transition for example, are needed, adiabatic methods are needed.^6^ Cyanine dyes, the focus of this work, have intense major peak consisting of low lying vibronic transitions appropriate for an adiabatic FC approach.

### Cyanine dyes

#### History and applications of cyanine dyes

**Fig. 1:**
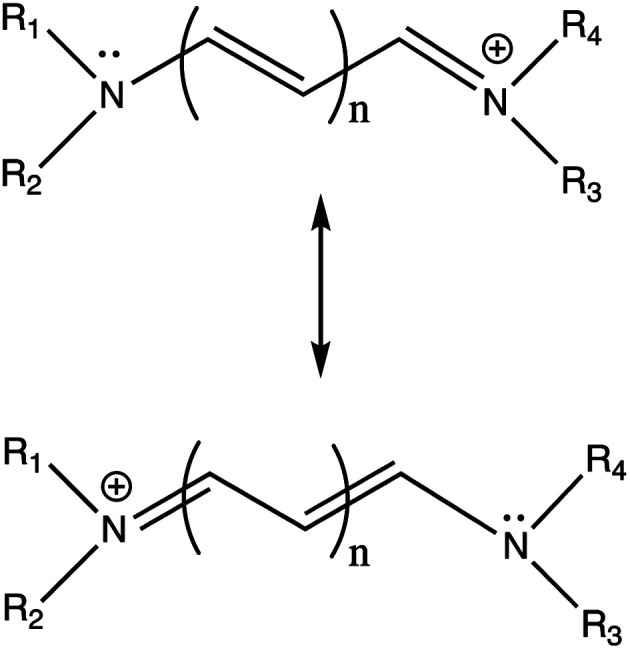
Prototypical cyanine dye resonance structures.

Cyanine dyes have a long history and have generated a great deal of interest both for their practical applications and as subjects of theoretical studies toward understanding the relationship between electronic spectra and molecular geometry. Mills and Wishart (1920) cite Spalteholz as having discovered cyanine dyes in 1883 independently of Hoogewerff and van Dorp also in 1883 and it is clear from Mills and Wishart's article that the molecular structure of cyanine dyes had become a subject of intense interest by 1920.^8^ The distinguishing feature of cyanine dyes is a conjugated carbon chain with nitrogen atoms on either end. This structural motif has an odd number of atoms and positive charge. Bond lengths tend to be symmetric on either side of the central carbon atom in the chain as predicted by use of two equally weighted resonance structures as shown in Fig. 1.

**Fig. 2:**
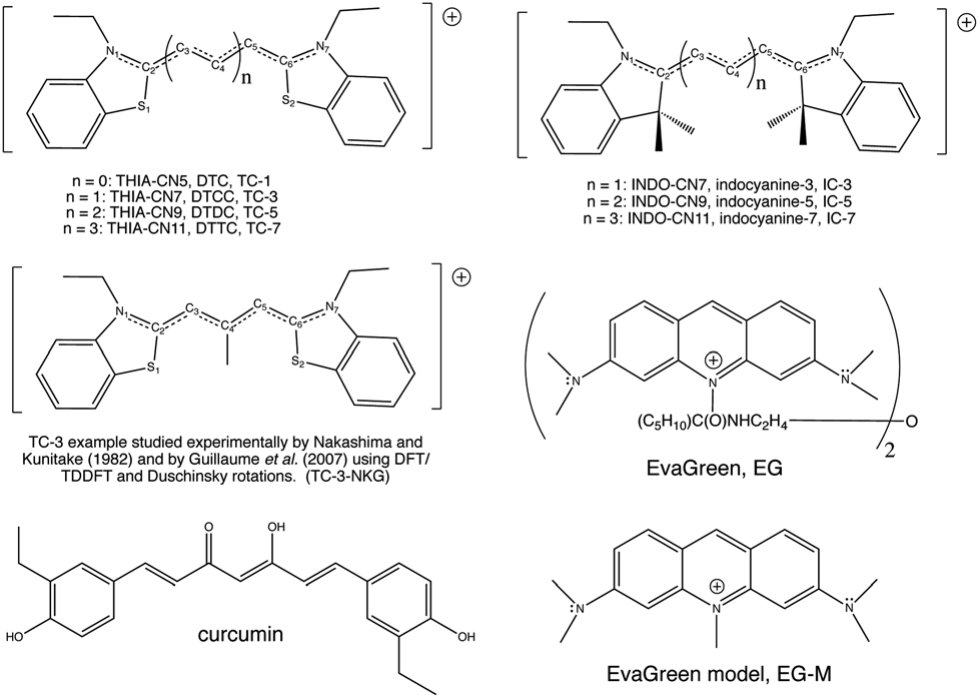
Molecular structures of 3,3′-diethylthiacyanine, 3,3′-diethylthiacarbocyanine, 3,3′-diethylthiadicarbocyanine, and 3,3′-diethylthiatricarbocyanine (top left), TC-3-NKG example (middle left), curcumin (bottom left), 1,1′-diethyl-3,3,3′,3′-tetramethylindocarbocyanine, 1,1′-diethyl-3,3,3′,3′-tetramethylindodicarbocyanine, 1,1′-diethyl-3,3,3′,3′-tetramethylindotricarbocyanine (top right), EvaGreen (middle right), a model of the EvaGreen fluorophore (bottom right). Common abbreviations are included. Hydrogen atoms bonded to the polymethine chain carbon atoms are not shown. Atom numbering shown here will be referred to in the text.

Cyanine dye model systems are referred to by the number of atoms in the conjugated system including nitrogen atoms. For example, if in Fig. 1*n* = 1 the dye is CN5 or if *n* = 2, the dye is CN7 *etc.* The nitrogen atoms are frequently part of heterocyclic ring end groups in which case the dyes are referred to by the number of carbon atoms in the conjugated chain between end groups. Examples that are the focus of this paper are shown in Fig. 2. THIA and INDO dyes and modifications based on them have applications in biomedical imaging. TC-1 shows enhanced fluorescence in the presence of bacterial cells.^9^ Nakashima and Kunitake (1982) found enhanced fluorescence of the TC-3-NKG example (Fig. 2 middle left) in the presence of aqueous lipid bilayers.^10^ TC-3 and TC-3-NKG show enhanced fluorescence in the presence of DNA relevant to the study of gene regulation,^11^ and an analogue of TC-3 and TC-3-NKG was shown to have fluorescence sensitive to oligomeric and fibrillar α-synuclein relevant to Parkinson's disease.^12^ TC-7 has been used in *in vivo* studies to quantify nerve cell myelination and to measure DNA helicity and sequence.^13,14^ Variations of IC-3 and IC-5 are used as covalent labeling dyes to monitor the budding and fusion events of viruses in living cells.^15^ These two indocyanine dyes have also been found useful in targeting mitochondria in cancer cells and delivering cargoes to them.^16^ EvaGreen, also shown in Fig. 2, displays enhanced and redshifted fluorescence when bound in the DNA minor groove and is an effective reagent for detection of autoimmune antibodies in qPCR methods.^17–19^ Both THIA and INDO dyes are subjects of study for use in DSSCs.^20,21^ We will refer to these dyes and their analogues with H or methyl substituted for ethyl on the nitrogen atoms according to abbreviations given in Fig. 2. Curcumin, also shown in Fig. 2 in its keto–enol form, is not a cyanine dye. Its seven-carbon conjugated chain and aromatic end groups give curcumin a similar structure to the THIA and INDO dyes. Electron delocalization contributing to curcumin's strong absorption at 425 nm and fluorescence at 542 nm in ethanol does not involve nitrogen atoms as in cyanine dyes. Moreover, curcumin has neutral charge and the oxygen substitution of its conjugated chain is a central feature contributing to its chemical and photophysical properties. Curcumin is included in this study to compare and contrast with results on cyanine dyes.

#### Theoretical approaches toward studying cyanine dyes

Bury (1935) recognized the intense absorption of cyanine dyes to be related to their accurate description by two equally weighted resonance structures as in Fig. 1.^22^ According to resonance theory, and assuming equal weighting of the two major resonance structures, one expects carbon–carbon and carbon–nitrogen bond lengths to be symmetric on either side of the central polymethine carbon rather than of alternating bond length as typical in linear polyenes and as would be indicated if one of these two major resonance structures were favored. Shorter chain length cyanine dyes have symmetric structures indicating a high degree of π-electron delocalization.

The π-system electron delocalization between the nitrogen atoms is the basis of the success of Kuhn's 1949 application of the particle-in-a-box model toward understanding the increase in absorption *λ*_max_ with increased number of carbons in the polymethine chain.^23^ Polymethine chain bond length alternation (BLA) broadens peaks in electronic spectra and can shift the position of *λ*_max_.

BLA in cyanine dyes with the polymethine chain length of seven or more carbon atoms has been observed under some circumstances and has been the subject of both experimental and theoretical interest. Ragni *et al.* (2019) found the large stokes shift of some C7 dyes to be explained by ground state BLA sensitive to substitution and the central carbon of the polymethine chain and solvent polarity followed by relaxation to a symmetric exited state.^24^ Gierschner *et al.* (2020) showed similar C7 cyanine dyes also with substitution at the central carbon to have BLA sensitive to counter ions in solution and in their crystal structures and found this to cause broadened and redshifted absorption spectrum.^25^ Crossover from symmetric to BLA structures depends on dye functionality, polymethine chain length and media. Yesudas (2013) found agreement between results using DFT (their custom density functionals and M06HF) and experiments showing that cyanine dyes, including the TC dyes shown in Fig. 2, are symmetric in solution and show BLA with chain lengths of eleven or more carbon atoms.^26^ Potenza *et al.* (1978) using X-ray crystallography found that TC-7 iodide crystals have BLA.^27^ The published literature indicates the dyes that are the focus of this article are symmetric in solution, even in polar solvents. This is an important consideration in regard to the accuracy of TDDFT results compared to experimental spectra as electronic and vibrational properties depend strongly on this symmetry.

Guillaume *et al.* (2007) using TDDFT and Franck–Condon analysis based on Duschinsky rotations found good agreement to the experimentally observed shoulders in absorption and emissions spectra of TC-3-NKG and vibrational assignments indicating these shoulders result from a collection of singly excited vibrations rather than a unique dominant vibrational mode as argued by Mustroph (2018).^28,29^ There does not seem to be disagreement that many low frequency vibrations contribute to the dominant band.^28,29^ The computations of Guillaume *et al.* (2007) are *in vacuo* and they conclude the remaining differences between their computed vibronic spectra and experiment are due to media and the limitation of the XC density functional.^28^

The limitations of a variety of density functional and wavefunction approaches applied to cyanine dyes have been the subject of a number of studies. Comparison between computed vertical transitions from a variety of TDDFT and wavefunction methods and with experiment for the simplest cyanine dye examples (R_1_ = R_2_ = R_3_ = R_4_ = H in Fig. 1) has been a topic of interest and debate. Send *et al.* (2011) concluded comparison of computed vertical energies to experiment is unreliable due to non-vertical transitions and cite Dierksen and Grimme (2004) who found Franck–Condon and Herzberg–Teller analysis based on TDDFT computations give results in good agreement with experiment for a number of polyene and aromatic examples.^30,31^ According to Send *et al.* (2011), vertical energies from quantum Monte Carlo, single reference and multi-reference wavefunction methods, and B2PLYP methods give reasonably close excitation values which are improvements on GGA, hybrid GGA, and meta hybrid GGA methods.^30^ Truhlar *et al.* (2012) subsequently showed these classes of TDDFT give errors in a similar range as wavefunction methods, 0.10–0.36 eV and 0.16–0.34 eV respectively, with respect to quantum Monte Carlo results which are considered to be the best estimate and found M06HF to be most accurate among the Minnesota density functionals for this application.^32^ Later work by Jacquemin *et al.* (2015) indicates TDDFT methods systematically overestimate cyanine dye excitation energies “irrespective of the details of the computational protocol” and continuing effort to accurately describe their photophysical properties. They report wavefunction methods to be more accurate but not feasible for “real-life” cyanines.^33^ Interest in practical applications of cyanine dyes has prompted computations beyond these simplest cases including the TC dyes studied here.

Cole *et al.* (2016) found vertical excitation energies for TC-3, TC-5, and TC-7 with H- and Me-substituted for the nitrogen ethyl groups and the TC-3 analogue with oxygen substituted for sulfur, calculated using TDDFT with the M06HF meta hybrid density functional overestimated excitation energy of 0.51 eV on average compared to experiment and improved to 0.35 eV with solvent effects included while LT-DF-DCC2 computations corrected for solvent effects gave vertical excitation energies greater than experimental *E*_abs_ by an average of 0.15 eV.^21^ We note that the error of 0.35 eV reported by Cole *et al.* using M06HF is smaller than that found using other functionals BHandHLYP for example.^25^ TDDFT methods are widely available and computationally accessible and this has motivated the use of empirical corrections to their results when applied to cyanine dyes.

**Fig. 3:**
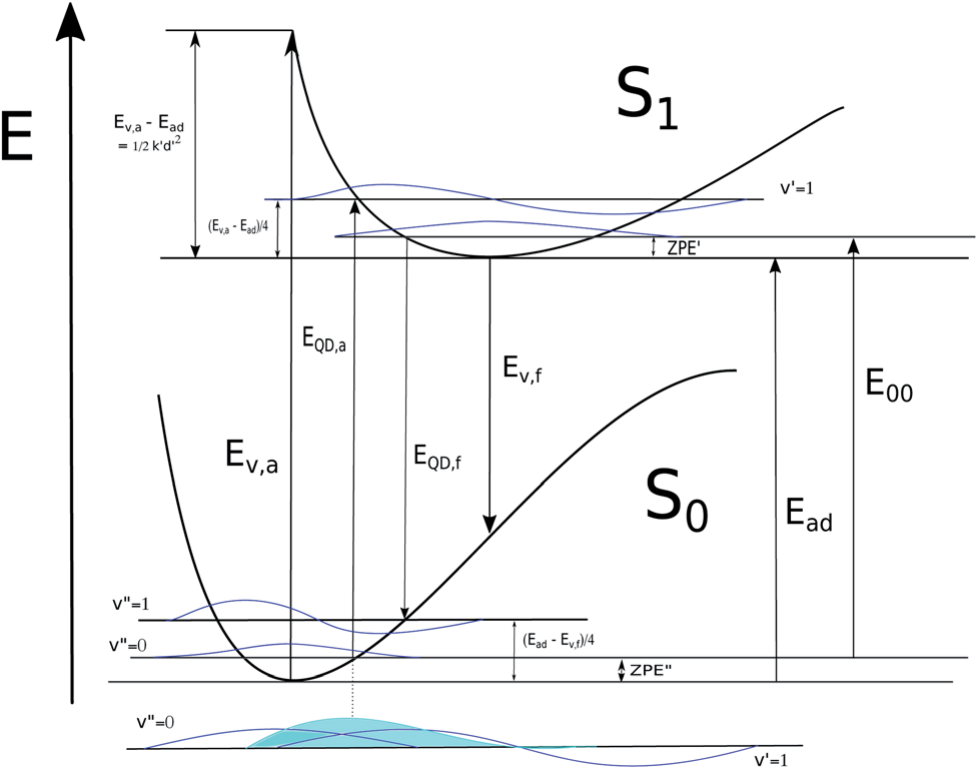
Depiction of energies associated with computed transitions for absorption and fluorescence and with the proposed quarter difference (QD) approximation of relaxation energies. The product of ground and excited state vibrational wavefunctions involved in a vibronic transition is depicted with blue shading. It is presumed in comparison to the r-centroid that the QD approximation corresponds to a location at or near the maximum of this product for vibronic transitions contributing to the absorption or fluorescence spectrum.

Spichty *et al.* (2011) devised a conceptually informed four-parameter empirical approach for predicting absorption wave-length of cyanine dyes from their computed TDDFT vertical energies, *E*_v,a_ in Fig. 3, and zero point vibrational energies.^34^ Nakano *et al.* (2019) compared *λ* from TDDFT *E*_v,a_ to experimental absorption *λ*_max_ for 70 polymethine dyes including the methyl analogues of TC-3, TC-5, TC-7, IC-3, and IC-5 and found strong linear relationships and also confirmed the systematic underestimation of *λ*_max_ when this comparison is made using a wide range of fourteen density functionals including GGAs, hybrid-GGAs, meta-GGAs, hybrid-meta GGAs, and a hybrid-meta-NGA.^35^ There is enough consistency in results across these functionals that the averaged regression curve from combined results can be used to quantitatively predict *λ*_max_. These empirical approaches are of practical value because they allow predictions to be made based on computed ground state properties without the more time consuming task of finding an optimized excited state geometry and carrying frequency analysis on it. Still, the nature of the systematic error in comparing computed *E*_v,a_ to experiment is not fully accounted for.

We propose the error resulting from comparison of TDDFT *E*_v,a_ to experimental *E*_abs_ = *hc*/*λ*_max_ is based, in part, on the fact that electronic transitions from an exactly specified molecular geometry are not a well-defined idea in the context of the quantum mechanical Franck–Condon principle.^3,4^ The computed vibronic spectra would then be expected to give improved accuracy in *E*_abs_ = *hc*/*λ*_max_ provided the band shape predicted for the major peak is reasonable. One might expect a vertical transition from an averaged molecular geometry to be an appropriate comparison to experimental spectra in cases where classical approximations are appropriate and classically vertical transitions conserve kinetic energy due to nuclear motion as Zare and Noda demonstrated for the r-centroid.^5^ The contribution of low frequency vibrations to the major band in cyanine dye electronic spectra may make them a class of molecules conducive to such a classical approximation for their most intense spectral peak.

In this work we perform Franck–Condon analysis on *N*-methyl versions of cyanine dyes in Fig. 2 to find vibronic absorption and emission spectra with solvents described by implicit solvation. Curcumin, a non-cyanine dye, is included for the sake of comparison. Computed absorption and emission *λ*_max_, Stokes shifts, and *E*_00_ are compared with those from experiment. The major peak in the computed vibronic spectra is found to consist of numerous low frequency transitions. A simple model is developed utilizing computed vertical energies from the optimized geometries of the initial electronic state, *E*_v,a_ and *E*_v,f_, for absorption and fluorescence respectively, the adiabatic energy, *E*_ad_ and zero-point vibrational energies of both states involved in the transition. These energies are depicted in Fig. 3. The approach is based on the harmonic approximation and the idea that a classical approximation would represent vibronic transitions as taking place vertically from an averaged molecular geometry. This simple model gives absorption and fluorescence energies with only slightly less accuracy than spectra computed from FC analysis and both methods are found to have high accuracy compared to experiment.

## Computational methods

### Geometry optimization and FC analysis

Methyl analogues of the THIA and INDO dyes, curcumin, and EG-M, in Fig. 2 were the subject of computations carried out using Gaussian 09, Revision B.01 (ref. 36) and Gaussian 16, Revision A.03 (ref. 37) software. B3LYP^38–41^ and M06HF^42^ hybrid and meta hybrid density functionals were used with the 6-31G(d) basis set for most examples. A wide variety of density functionals have been used in TDDFT studies of cyanine dyes and with consistent overestimation of vertical energies as discussed in the introduction. B3LYP and M06HF are among the functionals found to give correct BLA predictions and by using them, we avoid the larger error in TDDFT vertical energy expected from BHandHLYP but not the overall systematic error in comparison of vertical energies from the optimized geometry of the initial electronic state to experimental *λ*_max_.^24–26^ BHandHLYP computations were carried out on TC-3-NKG using 6-31G(d), 6-311G(d), and SVP basis sets for the sake of comparing to results in the literature. We found *E*_v,a_ and *E*_v,f_ for curcumin to be far more sensitive to % HF exchange than for cyanine dyes and we found M06 to give the most accurate results for this case.

In choosing density functionals for the purpose of computing vibrationally resolved spectra of dye molecules one should keep in mind that band shape can have an effect on *λ*_max_ and functionals that predict accurate band shape in vibronic spectra are not necessarily well suited for predicting accurate overall position in the spectrum. In their study of coumarin dyes, Bloino *et al.* (2015) found use of two functionals, ωB97x for accurate band shape shifted by PBE0 wavelengths for accurate position in the spectrum, gave best agreement with experiment.^7^ They found PBE0, M06, and B3LYP functionals to result in vibronic spectra drastically underestimating the intensity of bands due to higher frequency vibrations. We found that for application to cyanine dyes in this study, B3LYP and M06HF give reasonably good vibronic band shape and position in the spectrum compared to experiment and to other functionals used in the literature. Perhaps the dominant peak in cyanine dyes near the 0–0 transition and broadened and shifted by low frequency transitions is less sensitive to choice of density functional. Our B3LYP and BHandHLYP calculations on TC-3-NKG and results reported in the literature on a wider range of cyanine dyes indicate predicted *λ*_max_ for this peak is fairly insensitive to choice of basis set.^34^

Ground and excited state molecular geometries were optimized in solution according to a polarization continuum model, PCM in Gaussian software packages used.^43–59^ Effects of nonequilibrium solvation were approximated using a linear response correction to absorption energies, *E*_v,a_, calculated from the ground state equilibrium geometry. The nonequilibrium linear response solvation method includes this solvation effect directly in the TDSCF vertical absorption energy for the specified CI-singles excited state.^55,60,61^ This option is not available for calculation of *E*_v,f_ for which equilibrium solvation was used.^60^ State specific nonequilibrium solvation, a method that entails iterative optimization of the final electronic state charge distribution and interaction with solvent based on the input solvent reaction field from the initial electronic state, was applied to TC-3 for the purpose of comparison.^62,63^ It was found that, for absorption, the linear response method gave results closer to experiment. Nonequilibrium state specific solvation for TC-3 emission was close to results using equilibrium solvation so it was concluded that nonequilibrium solvation effects are small for emissions processes for this example.

Ground state and excited state geometries were optimized using “tight” convergence and ultrafine grid integration criteria. Local minima in ground electronic state geometries were confirmed with all real vibrational frequencies. Vertical excitation energies, *E*_v,a_, were computed from the optimized ground state geometries using TDSCF with the same density functionals, integration, and solvent as used for the ground state geometry optimization and corresponding geometry optimization of the lowest energy singlet excited state. Local minima were verified with all positive real vibrational frequencies for all examples except TD M06HF for TC-5 and TC-7 for which two imaginary frequencies remained. The problematic vibrational modes for these two cases involved symmetric and antisymmetric rotation of the methyl groups coupled to lowest wavenumber symmetric and antisymmetric flexing modes of the polymethine chain. Adjustment in geometry and recalculation were not successful in removing these imaginary frequency modes and these results could not be used for FC analysis. The M06HF ground state optimized geometry of TC-7 showed BLA in contrast to the observed experimental spectra of this dye in solution so these computational results were not used for further analysis.^64^

Methods of Barone *et al.* (2007–2009) were used to carry out time-independent Franck–Condon analysis corresponding to temperature of 0 K.^65–68^ Vibrationally resolved spectra were computed for the absorption spectrum of TC-3 based on M06HF results and absorption and emission spectra for TC-5, TC-7, and IC-5 using B3LYP results. *In vacuo* computations were performed for TC-3-NKG in order to compare with results of similar calculations in the literature.^28^ Vibronic spectra were computed for curcumin *in vacuo* but did not achieve sufficient spectral progression for curcumin in ethanol. Time dependent FC analyses were computed at temperatures of 0 K and 298.15 K.

### ONIOM model of TC-1 and EG-M in the DNA minor groove

A two-base-pair model of DNA with a backbone consisting five ribose phosphate diesters was constructed to approximate the environment of the DNA minor groove. Two magnesium dications octahedrally solvated with six water molecules each were used to partially balance the charge of the five phosphate diesters so the total charge of the DNA fragment was −1. The TC-1 and EG-M dyes have charge of +1 so the entire model had neutral charge. The dyes were placed in the minor groove and the complex was optimized using UFF^69^ and QEq^70^ charges on atoms before ONIOM^71–74^ calculations were carried out. The ONIOM calculations had 2 layers. The DNA layer was low and used UFF and the high layer used B3LYP/6-31G(d). The TC-1 excited state twisted and puckered indicative of a biradical state similar to the first excited electronic state of ethylene.^75^ MN15 was then used because it is parameterized for use in both single and multireference circumstances.^76^ A less twisted ES geometry resulted using MN15. B3LYP/6-31G(d) optimization of EG-M in the DNA minor groove model gave a geometry with two excited states close in energy, also an indication that MN15 may be a more appropriate method for this case. Optimization using MN15 resulted in a different excited state geometry without other nearby excited states. Both B3LYP and MN15 optimizations of EG-M excited state in the DNA minor groove gave a negative vibrational frequency precluding Franck–Condon analysis.

### The classically inspired QD model

#### Definitions and basic application

A model is developed here with the goal of predicting *λ*_max_ for the lowest frequency band in cyanine dye or similar dye molecules based on a classically vertical transition from a geometry averaged between those of the final and initial electronic states. The r-centroid approach is similar as a classical approximation based on an averaged geometry but has been applied to diatomic molecules. To extend a similar approach to molecules with more than two atoms, the current model is defined in terms of the Duschinsky transformation as follows.

(1) Duschinsky shift vector, **K**, in distance coordinates rather than mass weighted coordinates, defines the displacement **d**, from the equilibrium geometry of the initial state to that of the final state.

(2) The sum of harmonic displacement energies is equal to *E*_v,a_ − *E*_ad_ for absorption and *E*_ad_ − *E*_v,f_ for fluorescence, that is, 
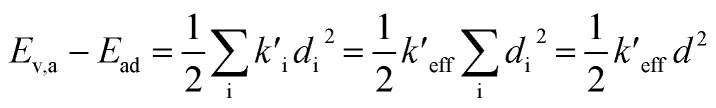
 for absorption and 
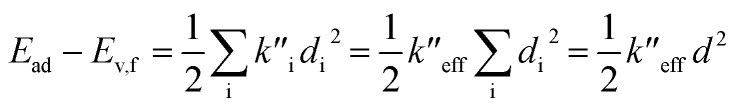
 for fluorescence.

(3) The position specified by **d**/2 is about equal to the vector sum of r-centroids along each mode.

(4) The energy difference between final and initial electronic state energies at **d**/2 corresponds to a classically vertical transition.

Though this classically inspired model is defined in terms of the Duschinsky transformation, which is generally an explicit part of AH and AS methods, the following equations show how the current model, also an adiabatic approximation requiring optimization of both electronic state geometries, is applied for estimation of *λ*_max_ without the use of **d** = **K** and the effective force constants discussed above. These, however, are useful for understanding what kinds of effects are included and where the model applies and will be discussed after its basic application is described. Our model can be applied in reference to *E*_ad_ or *E*_00_ but the results are better if ZPE energies are accounted for in ground and excited states so *E*_00_ is preferable if available.

1 *E*_00_ = *E*_ad_ − ZPE′′ + ZPE′

From the harmonic approximation the geometry at displacement **d**/2 would have energy above the equilibrium geometry of the final electronic state as shown be eqn (2) and (3). See Fig. 3.2
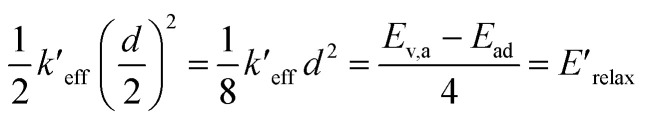
3
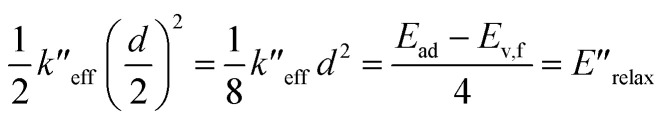


Eqn (2) and (3) are estimates of relaxation energy after excitation or after emission and allow calculation of *E*_QD,a_ and *E*_QD,f_ in eqn (4) intended as estimates of absorbance and fluorescence energies. See Fig. 3.4
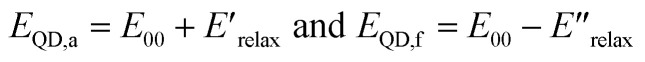
QD here stands for “quarter difference” in reference to eqn (2) and (3) and will be used to refer to this method through the rest of this paper.

#### The QD model compared to AH and AS approximations

Adiabatic models are preferable to vertical in cases where low frequency transitions near 0–0 are of interest and this is the case where classical approximations like the QD model apply. The physical picture of the QD model entails a strong correspondence between normal modes in ground and excited states for which the Duschinsky rotation matrix would be roughly diagonal. The model applies, therefore, for rigid molecules as have been subjects of adiabatic FC approximations. Displacement in the QD model is the Duschinsky shift vector, **K**, which depends on the optimized geometries of the final and initial electronic states and their respective normal modes and not on whether the rotation matrix, **J**, is strictly diagonal. A roughly diagonal rotation matrix is sufficient according to the physical picture espoused to the QD model.

**Fig. 4:**
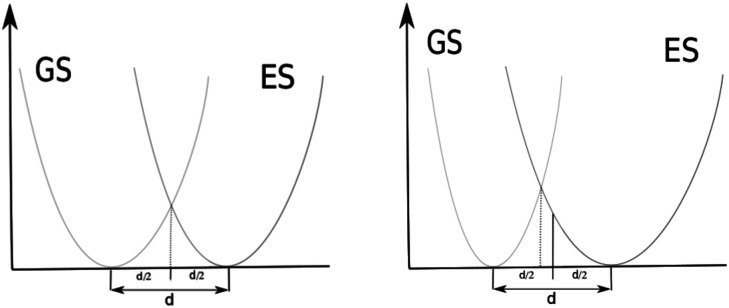
Intersecting harmonic potentials corresponding to AS models (left) and AH models (right).

The QD model corresponds most closely to an AS approximation as PES curves that are the same only displaced intersect at *d*/2. See Fig. 4 (left). Their ZPEs in this case are equal and therefore they have equal total vibrational energy in their *v* = 0 states. With total vibrational energy and total vibrational potential energy both equal, their total vibrational kinetic energy must also be equal, *i.e.* vibrational kinetic energy is conserved in a classically vertical transition from this position. If the harmonic potential of one electronic state is more narrow than the other, the point of intersection between the potentials shifts toward the minimum of the narrower potential. See Fig. 4 right. In this case, the ZPE of the state with the narrower potential is greater. The two states have the same vibrational potential energy at their intersection but the state with narrower potential has greater total vibrational energy in its *v* = 0 state and therefore greater vibrational kinetic energy according to the virial theorem. Moving back toward *d*/2, the classical vibrational potential energy increases so the classical kinetic energy must decrease for the narrower potential. This shift toward *d*/2 in the broader potential causes classical potential energy to decrease and classical kinetic energy to increase. The position at which classical vibrational kinetic energy is the same for both states is shifted back toward *d*/2. Transitions from *v* = 0 in the state with the narrower potential to overtones in the wider potential would be at classical positions with less shift from *d*/2 in order to have kinetic energy conserved in a classically vertical transition. Though the QD model shows a more direct correspondence to AS approximations for their 0–0 transitions, comparison of the QD model to AH approximations seems reasonable. We will refer to *E*_QD_ that use *E*_ad_ as the reference as *E*_QD_ − AS and those that use *E*_00_ as the reference as *E*_QD_ − AH. *E*_QD_ − AS will still include some effects of different GS and ES potential energy surfaces from definition (2) in the previous subsection.

#### The QD model, solvent, and anharmonicity

Relaxation energies in the QD model are estimated according to *E*_v,a_ − *E*_ad_ for absorption and *E*_ad_ − *E*_v,f_ for fluorescence. When solvent is included, *E*_v,a_ includes effects of nonequilibrium LR solvation and *E*_ad_ and *E*_v,f_ include effects of equilibrium solvation. This means that the same approximation is made in *E*_QD,a_ for solvent as solute, and effects of nonequilibrium and equilibrium solvation are averaged, appropriate for longer time-scales for which low frequency vibrations are resolved. LR solvation applies where solvent energies follow a Gaussian distribution as described by a bath of classical oscillators appropriate for the classical picture in the QD model. The solvent approximation implicit in *E*_QD,f_ is based on equilibrium solvation for both final and initial electronic states. The fluorescence redshift may mean that equilibrium solvation is more appropriate for fluorescence. As mentioned in the Computational methods section, nonequilibrium solvation effects appear to be small for fluorescence of the TC-3 cyanine dye and it seems this would also be the case for other dyes studied here.

*E*_QD,a_ implicitly includes nonequilibrium effect of solvent and according to the definitions enumerated according to the Duschinsky transformation, this means the effective force constant, 
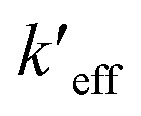
, absorbs this effect. PCM solvation has an effect on computed vibrations and not all vibrations will be effected in the same way. Normal modes with greater displacement in the Duschinsky vector have greater contribution to the vibronic spectrum and are weighted more in their contribution to *E*_v,a_ − *E*_ad_ or *E*_ad_ − *E*_v,f_ therefore the effect of PCM solvation on vibrations contributing to the vibronic spectrum is also implicit in the QD model. Solvent effects cause initial and final electronic states to have different equilibrium geometries than they do *in vacuo* causing the displacement to be different and likely greater in which case the effects of anharmonicity would be greater in solvent. These differences are also absorbed into energy differences *E*_v,a_ − *E*_ad_ or *E*_ad_ − *E*_v,f_ and absorbed into the QD model through the effective force constants given that the anharmonicities do not result in significantly different mode mixing than predicted by use of the harmonic approximation at ground and excited state equilibrium geometries. This is more likely true for rigid or semi-rigid molecules.

## Results and discussion

### Accuracy of absorption and fluorescence energies from computed vibronic spectra

Computed vertical excitation and emission energies, *E*_v,a_ and *E*_v,f_ compared to *E*_abs/fl_ = *hc*/*λ*_max_ from experiment are presented in Table 1. The mean error in comparison of *E*_v,a_ and *E*_v,f_ to experimental *E*_abs/fl_ = *hc*/*λ*_max_ is 0.4 eV for absorptions and 0.2 eV for fluorescence. These errors are consistent with the literature and somewhat smaller than that reported in some cases. Gierschner *et al.* (2020) for example report 0.5 eV in their TDDFT application of BHandHLYP/6-311G*.^25^

**Table 1:** Computed *E*_v,a/f_ compared to *E*_abs/fl_ = *hc*/*λ*_max_ from experiment. Energies in eV

Dye	*E*_v,a_ comp.	*E*_abs_ exp.	*E*_v,f_ comp.	*E*_fl_ exp.
TC-3 ethanol	2.652^a^	2.220^e^	2.198^c^	2.170^e^
TC-5 water	2.319^b^	1.91^f^	1.912^d^	
TC-5 ethanol	2.304^b^	1.891^e^	1.931^d^	1.831^e^
TC-7 water	2.075^b^	1.647^f^	1.682^d^	
TC-7 methanol	2.077^b^	1.638^f^	1.695^d^	1.553^g^
TC-7 2-propanol	2.057^b^	1.642^e^	1.710^d^	
TC-7 DMSO	2.043^b^	1.605^f^	1.688^d^	1.52^g^
IC-5 methanol	2.353^b^	1.945^e^	1.956^d^	1.887^e^
Mean error	0.4 ± 0.2		0.1 ± 0.06	

a M06HF/6-31G(d) and PCM nonequilibrium linear response solvation.

b B3LYP/6-31G(d) and PCM nonequilibrium linear response solvation.

c M06HF/6-31G(d) and PCM equilibrium solvation.

d B3LYP/6-31G(d) and PCM equilibrium solvation.

e PhotochemCAD^77–79^https://www.photochemcad.com.

f West and Pearce (1965).^80^

g Sorokin *et al.* (1967).^64^

**Fig. 5:**
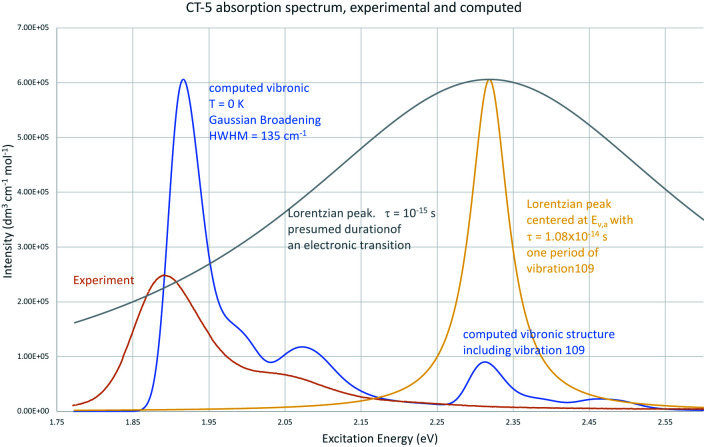
Experimental absorption spectrum of CT-5 in ethanol (red) compared to its computed vibronic spectrum (blue), Lorentzian peak corresponding to a presumed duration for an electronic transition of 10^−15^ s (grey), and a Lorentzian peak centered at *E*_v,a_ and corresponding to the period of a computed vibration corresponding to the nearest vibronic peak.

*E*_abs/fl_ = *hc*/*λ*_max_ from the major peak in the computed vibronic absorption and emissions spectra show better agreement with those from experimental spectra and give mean errors of 0.04 eV for absorption and 0.05 eV for fluorescence. See Table 2. We believe the high degree of accuracy of *E*_abs/fl_ = *hc*/*λ*_max_ from our computed vibronic spectra compared to experiment as opposed to *E*_v,a_ and *E*_v,f_ which are more frequently compared in the literature is due to the error in comparing the vertical energy from the optimized geometry of the initial electronic state to experimental *E*_abs/fl_ = *hc*/*λ*_max_. Our computed vibronic spectra allow us to predict where and if vertical energies from the equilibrium geometry may occur in the Franck–Condon region and how well they compare to computed and experimental vibronic peaks. See Fig. 5.

**Table 2:** *E*_abs/fl_ = *hc*/*λ*_max_ from computed vibronic spectra compared to those from experimental spectra. Energies in eV

Dye	*E*_abs_ comp.	*E*_abs_ exp.	*E*_fl_ comp.	*E*_fl_ exp.
TC-3 ethanol	2.231^a^	2.220^d^		2.170^d^
TC-5 water	1.916^b^	1.91^e^		1.879^c^
TC-5 ethanol	1.932^b^	1.891^d^	1.894^c^	1.831^d^
TC-7 water	1.683^b^	1.647^e^	1.636^c^	
TC-7 methanol	1.694^b^	1.638^f^	1.587^c^	1.553^f^
TC-7 2-propanol	1.708^b^	1.624^d^	1.663^c^	
TC-7 DMSO	1.668^b^	1.605^f^	1.645^c^	1.52^f^
IC-5	1.962^b^	1.945^f^	1.935^c^	1.887^f^
Mean error	0.04 ± 0.02		0.05 ± 0.05	

a M06HF/6-31G(d) and PCM nonequilibrium linear response solvation, time-independent FC analysis at 0 K.

b B3LYP/6-31G(d) and PCM nonequilibrium linear response solvation, time-independent FC analysis at 0 K.

c B3LYP/6-31G(d) and PCM equilibrium solvation, time-independent FC analysis at 0 K.

d PhotochemCAD https://www.photochemcad.com.^77–79^

e West and Pearce (1965).^80^

f Sorokin *et al.* (1967).^64^

An experimental spectrum of CT-5 in ethanol is shown in Fig. 5 along with its computed vibronic absorption spectrum.^77–79^ The position of the vertical excitation energy from the ground state equilibrium geometry, *E*_v,a_, is shown as the maximum of a Lorentzian peak with width based on the period of a vibration contributing to the nearest computed vibronic peak, mode 109, a C–H stretching mode on the *N*1 and *N*7 methyl groups. Higher frequency vibrations have shorter periods of vibration and would therefore be shown as wider Lorentzian peaks. A fluorescence lifetime on order of picoseconds, typical for cyanine dyes, would allow for about 100 periods of this vibration so an appropriate natural line width would likely be narrower. The width of the corresponding computed vibronic peak as displayed is based on the large number of predicted vibronic transitions contributing to it and Gaussian broadening of 135 cm^−1^, a default used by the GaussView 6.1 program. There are two goals to showing *E*_v,a_ in this way (1) to demonstrate that *E*_v,a_ is in the Franck–Condon region of the computed vibronic spectrum but does not correspond to vibrational structure that agrees well with *λ*_max_ in the experimental spectrum and, (2) to illustrate the arguments of Schwartz (1973),^4^*i.e.*, lifetimes on order of the period of molecular vibrations are consistent with vibronic peaks whereas measurements based on a presumed duration for an electronic transition, 10^−18^ s to 10^−15^ s do not allow sufficient resolution to show vibronic peaks as shown by the Lorentzian curve also centered at *E*_v,a_ but corresponding to a lifetime of 10^−15^ s. Shorter lifetimes result in even broader peaks; a lifetime of 10^−18^ s would appear as a horizontal line. The duration of an electronic transition is neither necessary nor well-defined in the context of the quantum mechanical version of the Franck–Condon principle. Specifying the position from which an excitation occurs as the equilibrium geometry of the initial electronic state and *E*_v,a/f_ as most appropriate for comparison to experiment is therefore not well-founded. The major peak shown in the computed vibronic spectrum in Fig. 5 results from vibronic transitions involving lower frequency vibrations and shows good agreement with experiment while peaks resulting from vibronic transitions involving higher frequency vibrations are not apparent in the experimental spectrum.

**Fig. 6:**
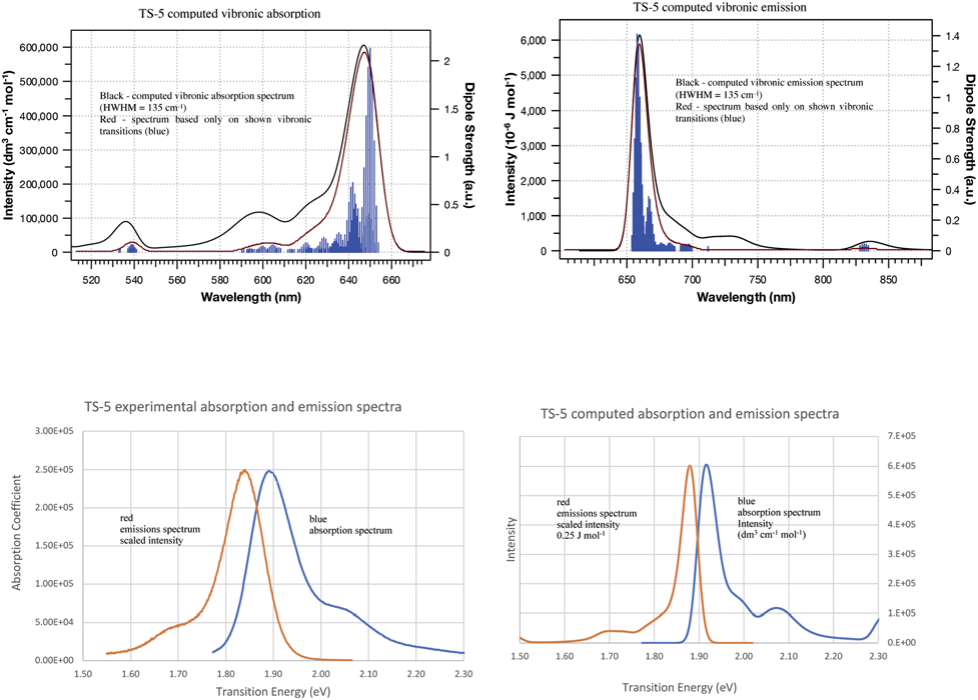
Vibronic absorption and emission spectra for TC-5: computed absorption spectrum (top left), computed emissions spectrum (top right), experimental absorption and emission spectra (bottom left), computed absorption and emissions spectra (bottom right).

Computed vibronic spectra for TC-5 are shown alongside experimental ones in Fig. 6.^77–79^ Vibronic transition sticks of 0.0005% intensity and greater in blue show how the contours of the computed spectra result from the predicted vibronic transitions. Agreement between the computed and experimental spectra suggests the contours in the experimental absorption and emissions spectra are due to vibrational structure. Intersection of the experimental absorption and emissions spectra indicates *E*_00_ = 1.87 eV, different from *E*_00_ = 1.91 eV from computations, 2.1% error. The vibronic transitions responsible for maximum intensity are close in energy to *E*_00_ and are due to low frequency breathing and bending modes in ground and excited state. The higher energy vibronic transitions are largely due to symmetric C–C stretching modes and the highest from C–H stretching modes. Duschinsky matrices for the examples studied here are an indication of the mixing of normal modes due to geometric distortion between ground and excited state geometries. These are largely diagonal indicating a small degree of mixing of normal modes. Many of the predicted vibronic transitions involve simultaneous vibronic excitation of two and in some cases three vibrational modes. These would seem to also be important in computed spectra at typical laboratory temperatures. Computational memory constraints precluded use of time-independent FC methods to compute vibronic spectra at 298 K.

### Computed spectra at 0 K and 298 K

**Fig. 7:**
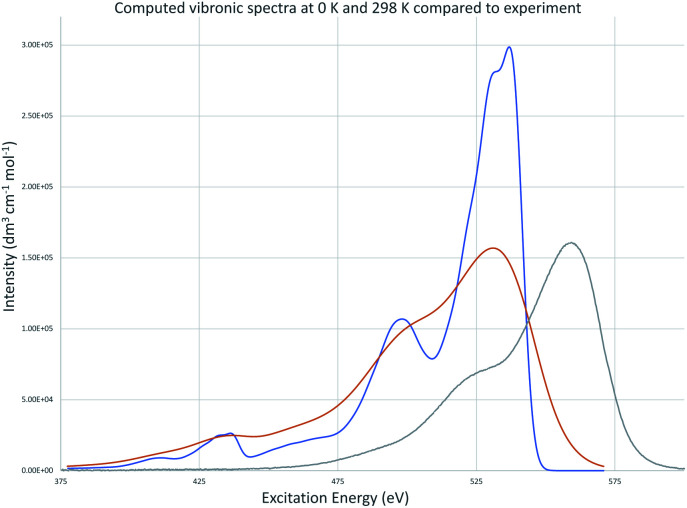
Absorbance of TC-3 in ethanol, computed vibronic spectrum at 0 K (blue), computed vibronic spectrum at 298 K (red), experimental spectrum (green). Computed spectra are shown with Gaussian broadening of 135 cm^−1^ width at half maximum.

Vibronic spectra were also computed using time-dependent FC analysis. This approach allowed prediction of spectra at temperatures higher than 0 K with less computational demand by reducing the amount of memory needed which made time-independent computations inaccessible to us. The large memory demands found for room temperature time-independent FC computations is likely due to the large number of initial state low frequency vibrational energy levels that are thermally accessible at 298 K. The vibronic transitions due to low frequency vibrations make the major contributions to the major peak for cyanine dyes and are the focus of the QD method so cannot be neglected for the purposes of this work. TD vibronic spectra at 0 K matched those computed using time-dependent FC analysis while those at 298 K had lower intensity and showed less well-defined vibrational structure giving a closer match to the intensity of peaks in experimental spectra. The absorbance spectra of TC-3 in ethanol are shown in Fig. 7 as an example. The contours of the computed TC-3 absorption spectrum shown in Fig. 7 are a good match to the experimental spectrum. This was true to varying degrees for the examples studied. Computed vibronic spectra at 298 K tended to overestimate intensity at higher/lower excitation/emission energy compared to experimental spectra from the literature. While temperature had a pronounced effect on intensity and appearance of vibrational structure in the computed spectra, effect on *λ*_max_ was negligible. The lower intensity with more thermally accessible vibrations at 298 K compared to 0 K in the computed spectra is suggestive of the enhanced fluorescence intensity of cyanine dyes with motions restricted by viscous media, aggregation, or binding to biomolecules.^9,10,17,81^

Time-dependent FC methods do not give assignments for vibronic transitions. The good agreement between time-dependent and time-independent FC spectra at *T* = 0 K and analogous appearance of the spectra at room temperature suggest the low frequency vibrations continue to make an important contribution to the spectra at 298 K. The major peak in the computed vibronic absorption and emissions spectra for the examples presented here involves low frequency transitions and are presumably amenable to application of classical concepts which do not entail vertical excitation from the equilibrium geometry of the initial electronic state but from a position resulting from a weighted average between states in the transition similar to the r-centroid, 〈**r**〉 = 〈*v*′|**r**|*v*′′〉/〈*v*′|*v*′′〉, as discussed by Noda and Zare.^5^ The QD method developed and applied here is drastically simplified in comparison, only requiring use of *E*_v,a/f_, *E*_ad_, and *E*_00_ and corresponds to an averaging between initial and final state optimized geometries. Results based on this approach are presented next.

### Results from the quarter difference method

Absorption and fluorescence energies calculated from the QD method are compared with experimental values in Table 3. QD-AH include the effect of different ZPE in initial and final electronic states and QD-AS do not. Mean errors included in the bottom row of Table 3 indicate both approaches are more accurate than the vertical energies from the equilibrium geometry of the initial electronic state (Table 1) and only slightly less accurate than predictions made using computed vibronic spectra, Table 2. QD-AH are more accurate than QD-AS which overestimate transition energies by an average of about 0.06 eV due to the fact that excited state ZPEs are less than ground state ones by this amount on average for these examples. The improved accuracy of *E*_QD,a_ from QD-AH compared to *E*_v,a_ is greater than *E*_QD,f_ compared to *E*_v,f_ which are only slightly more accurate. Both *E*_abs/fl_ = *hc*/*λ*_max_ from the computed vibronic spectra and the results from the QD method show excellent accuracy compared to results we have found in the literature from similar examples.^21,28^

**Table 3:** Computed *E*_QD,a/f_ compared to *E*_abs/fl_ = *hc*/*λ*_max_ from experimental spectra. Energies in eV

Dye/solvent	*E*_QD,a_ − AH	*E*_QD,a_ − AS	*E*_abs_ exp.	*E*_QD,f_ − AH	*E*_QD,f_ − AS	*E*_fl_ exp.
TC-1 water	2.975^h^	3.056^h^	2.917^i^	2.810^j^	2.890^j^	2.638^i^
TC-1 DNA	3.044^k^	3.152^k^		2.948^k^	3.055^k^	
TC-3 ethanol	2.286^b^	2.367^b^	2.220^e^	2.198^d^	2.270^d^	2.170^e^
TC-3 ethanol	2.388^a^	2.386^a^	2.220^e^	2.247^c^	2.273^c^	2.170^e^
TC-3 water	2.377^b^	2.357^b^		2.242^f^	2.2534^f^	
TC-3 water	2.377^a^	2.375^a^	2.242^f^	2.256^c^	2.253^c^	
TC-3-NKG *in vacuo*	2.587^l^	2.672^l^	2.583^m^	2.551^l^	2.668^l^	2.555^m^
TC-3-NKG water	2.295^b^	2.369^b^	2.292^n^	2.195^d^	2.270^d^	
TC-5 water	1.985^b^	2.051^b^	1.91^f^	1.896^d^	21.873^d^	
TC-5 ethanol	1.993^b^	2.062^b^	1.891^e^	1.900^d^	1.968^d^	1.831^e^
TC-7 water	1.747^b^	1.808^b^	1.647^f^	1.649^d^	1.710^d^	
TC-7 methanol	1.756^b^	1.819^b^	1.638^g^	1.660^d^	1.723^d^	1.553^g^
TC-7 2-propanol	1.761^b^	1.825^b^	1.624^g^	1.660^d^	1.739^d^	
TC-7 DMSO	1.743^b^	1.805^b^	1.605^g^	1.654^d^	1.716^d^	1.523^g^
IC-3 methanol	2.305^b^	2.397^b^	2.279^e^	2.192^d^	2.284^d^	2.218^e^
IC-5 methanol	2.028^b^	2.107^b^	1.945^e^	1.929^d^	2.008^d^	1.884^e^
IC-7 ethanol	1.800^b^	1.870^b^	1.667^e^	1.712^d^	1.783^d^	1.610^e^
EG-M water	2.533^b^	2.640^b^	2.638^o^	2.463^d^	2.569^d^	2.362^o^
Curcumin *in vacuo*	2.904^p^	3.024^p^	2.883^p^	2.847^p^	2.968^p^	2.869^p^
Mean error	0.08 ± 0.03	0.14 ± 0.01		0.07 ± 0.08	0.14 ± 0.02	

a M06HF/6-31G(d) and PCM nonequilibrium linear response solvation.

b B3LYP/6-31G(d) and PCM nonequilibrium linear response solvation.

c M06HF/6-31G(d) and PCM equilibrium solvation.

d B3LYP/6-31G(d) and PCM equilibrium solvation.

e PhotochemCAD https://www.photochemcad.com accessed July 2020.^71–73^

f West and Pearce (1965).^80^

g Sorokin *et al.* (1967).^64^

h MN15/6-31G(d) and PCM nonequilibrium linear response solvation.

i Thomas *et al.* (2010).^9^

j MN15/6-31G(d) and PCM equilibrium solvation.

k 2-Layer ONIOM MN15 for dye and UFF for DNA minor groove model.

l B3LYP/6-31G(d) no solvent model used.

m *E*_abs_ = *hc*/*λ*_max_ from B3LYP/6-31G(d) computed vibronic spectrum also *in vacuo*.

n Nakashima and Kunitake (1982).^10^

o Xin *et al.* (2007).^18^

p *E*_abs/fl_ = *hc*/*λ*_max_ from M06/6-31G(d) *in vacuo*.

To study these effects of solvent and anharmonicity in more detail we compared harmonic displacements energies from the computed normal modes and elements of the Duschinsky shift vector for TC-5 absorption and emission in the gas phase and in PCM ethanol and for curcumin in PCM ethanol and compared these with *E*_v,a_ − *E*_ad_ and *E*_ad_ − *E*_v,f_ on which the QD model is based. For TC-5 absorbance in ethanol *E*_v,a_ − *E*_ad_ = 0.323 eV and 
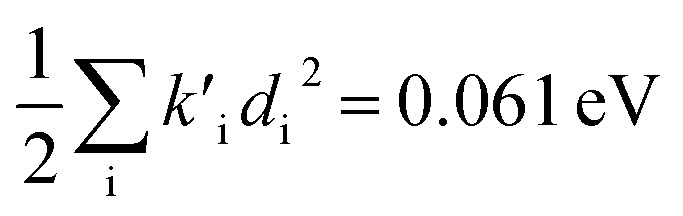
 indicate a large effect due to solvent not included in the computed normal modes. For TC-5 fluorescence in ethanol *E*_ad_ − *E*_v,f_ = 0.050 eV and 
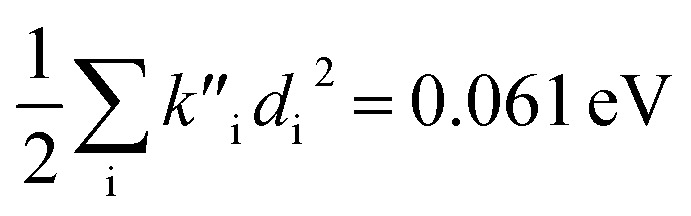
 were found, a much smaller difference. For TC-5 *in vacuo E*_v,a_ − *E*_ad_ = 0.0435 eV and 
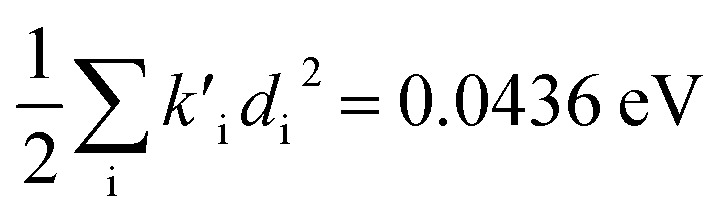
 were found for absorbance and *E*_ad_ − *E*_v,f_ = 0.0453 eV and 
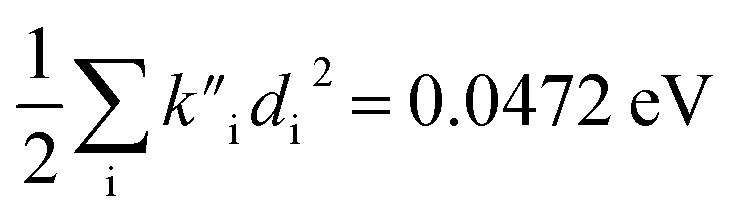
 for fluorescence. It is unclear how much of the difference between these TC-5 absorption results in ethanol and in vacuum are due to nonequilibrium solvation and how much due anharmonicity from the larger displacement in ethanol. For TC-5, rms displacement in ethanol is 1.71 Å while *in vacuo* it is 0.799 Å. The error for QD-AH for TC-5 in ethanol is 0.102 eV compared to 0.041 eV from the computed vibronic spectrum both typical of these methods applied to the molecules studied here. To see if the agreement *in vacuo* would be equally as close for a similarly shaped molecule but not a cyanine dye, the same approach was used for curcumin *in vacuo* resulting in *E*_v,a_ − *E*_ad_ = 0.112 eV and 
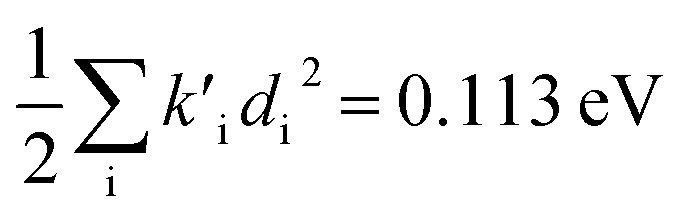
 for absorption and *E*_ad_ − *E*_v,f_ = 0.115 eV and 
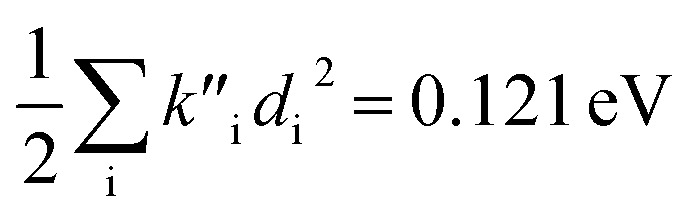
 for fluorescence.

**Fig. 8:**
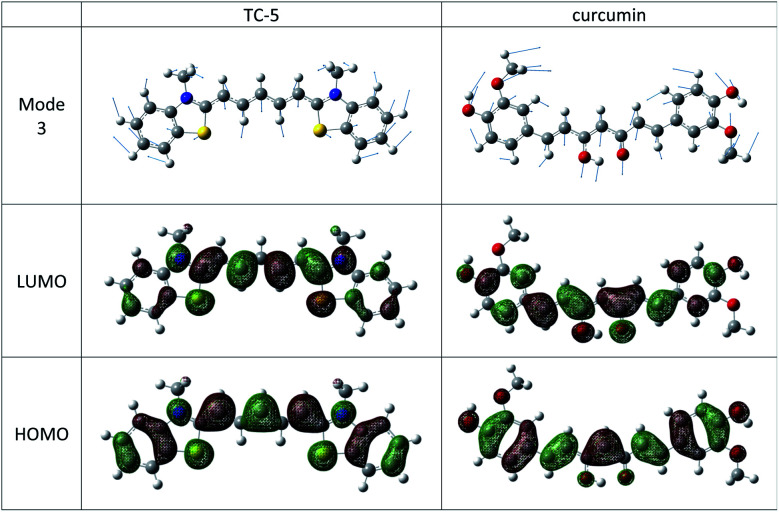
Mode 3 depicted for TC-5 and curcumin makes strong vibronic contribution to the major peak for these dyes. Its contribution along the displacement between GS and ES geometries is understandable according to frontier MOs.

The THIA and INDO cyanine dyes and curcumin have similar molecular structures, a conjugated carbon chain with aromatic end groups on either end. These examples have the same low frequency vibration, mode 3 with wavenumber of about 22 cm^−1^ that makes the most significant vibronic contribution to the major peak and has the largest Duschinsky shift contribution. Mode 3 in these cases consists of a flexing motion with the middle of the conjugated chain and end groups moving in opposite directions. See Fig. 8. Movement along this mode from initial to final state equilibrium geometries is understandable based on the phase of the HOMO and LUMO for the electronic transitions studied here which are well characterized as HOMO–LUMO. Contour plots of these orbitals for TC-5 and curcumin are also shown in Fig. 8.

Mode 1 in EG-M (36 cm^−1^) has a similar motion but out of plane, a motion that would seem to make a strong contribution along the path from ground to first excited state. A vibronic spectrum was not obtained for EG-M but errors using the QD model were similar to other examples except *E*_QD,a_ underestimates *E*_abs_ compared to experiment rather than overestimating it as in the other examples. This is an indication that higher frequency modes may make a stronger contribution to the vibronic lineshape of the dominant peak in EG-M. When EvaGreen is complexed to λDNA a longer wavelength peak which is only a shoulder in the spectrum of free EvaGreen becomes the dominant peak.^18^*E*_QD,a_ = 2.53 eV for EG-M is close to this lower energy peak that emerges when EG is complexed with λDNA with experimental *E*_abs_ = *hc*/*λ*_max_ = 2.50 eV. By analogy to other cyanine dyes, the higher frequency dominant peak in EG may be due to vibrations of *N*-substituents, the tether in the case of EG, and these may be damped when the two EG-M moieties are complexed in the DNA minor groove.

FC analysis for curcumin in ethanol did not result in a vibronic spectrum due to poor spectral progression. *E*_v,a_ is very sensitive to % HF exchange in the XC functional used for this example. M06 gave the best *E*_v,a_ and *E*_v,f_ values of the functionals tried which were not exhaustive, 2.93 eV (423 nm) compared to 2.92 eV (425 nm) from experiment for absorption and 2.47 eV (502 nm) compared to 2.287 eV (542 nm) from experiment for fluorescence.^77–79^ The large predicted fluorescence redshift is explainable by a shift of electron density onto keto–enol oxygen atoms causing pronounced stabilization by the PCM ethanol. The even larger fluorescence redshift found in experiment is ascribed to solvent dynamics involving solvent molecule disruption of the enol–keto H-bond leading to nonradiative decay and coupling between dark and emissive excited states resulting in longer emitted wavelength.^82^ Experimental absorption and fluorescence spectra of curcumin in ethanol at room temperature show broad peaks with no apparent vibronic structure.^77–79^ Application of the QD method using the M06–PCM ethanol results gave large underestimation of *E*_abs_ = *hc*/*λ*_max_, with an error of −0.307 eV. This error will be analysed in the next section.

### Accuracy of the QD method for cyanine dyes and limitations in extending application to other classes of molecules

The accuracy of *λ*_max_ from computed vibronic spectra and the corresponding predictions from the QD model for cyanine dyes and curcumin *in vacuo* highlight cases where these methods can be applied with accurate results. The example of curcumin in ethanol highlights effects that need to be considered when applying the model to other classes of molecules.

The results of FC analysis and the QD-AH method when applied to the THIA and INDO dyes are accurate when compared to experimental *E*_abs/fl_ = *hc*/*λ*_max_ with mean errors less than 0.1 eV, a much higher degree of accuracy than results when vertical energies from the equilibrium geometry of the initial state, *E*_v,a/fl_, are compared to experimental *E*_abs/fl_ = *hc*/*λ*_max_. *In vacuo* computations on TC-3-NKG were performed using BHandHLYP and SVP, 6-31G(d), and 6-311G(d) basis sets to compare with results from the literature and to investigate improvements gained from use of a solvent model as well a possible cancellation of errors which may be contributing to the accuracy of the results.

Our computed vibronic spectrum of TC-3-NKG *in vacuo* are similar to the results Guillaume *et al.* (2007) who found *E*_v,a_ of 3.01 eV, *E*_ad_ of 2.93 eV (423 nm) and *E*_00_ of 2.84 (437 nm).^28^ Guillaume *et al.* concluded the remaining error to be due to the media, methanol 2.29 eV (542 nm) or bilayer membrane 2.19 eV (565 nm), and the XC density functional. We performed BHandHLYP/SVP computations *in vacuo* to confirm Guillaume *et al.*'s results and then used BHandHLYP/SVP in PCM methanol to distinguish errors ascribable to this functional and to media. We found *E*_v,a_ of 2.87 eV, *E*_ad_ of 2.53 eV and *E*_00_ of 2.48 (499 nm) showing marked improvement when solvent is accounted for. Our results using B3LYP/6-31G* gave *in vacuo* results of *E*_v,a_ = 2.75 eV, *E*_ad_ of 2.69 eV, and *E*_00_ of 2.57 eV improved to *E*_v,a_ = 2.62 eV, *E*_ad_ of 2.28 eV, and *E*_00_ of 2.21 eV in PCM water in closer agreement with experimental *E*_abs_ = *hc*/241 nm of 2.29 eV with water as the solvent. B3LYP/6-31G* gives markedly more accurate results than BHandLYP/SVP so far as *E*_v,a_, *E*_ad_, and *E*_00_ are appropriate for comparison to experimental spectra.

*E*_QD,a_, calculated from our B3LYP/6-31G* results further improves accuracy predicting *E*_abs_ = 2.30 eV, 0.13% error compared to experiment, small compared to our average from Table 3, 3.8%, which is still less than the error form BHandHLYP/SVP in PCM methanol, 9.2%.

To investigate the effects of solvent in more detail, state specific nonequilibrium solvation methods were applied to TC-3 in ethanol resulting in an increase in *E*_v,a_ = 2.79 eV compared to the nonequilibrium linear response model *E*_v,a_ = 2.60 eV using B3LYP. Results were similar using M06HF. If the linear response solvation method underestimates effects of nonequilibrium solvation on excitation energies by about 0.2 eV, this would put the errors in excitation energies from FC and QD methods more in the range one expects for M06HF applied to C3, C5, and C7 cyanine dyes.^32^ It's possible some of the accuracy of FC and QD results presented here is due to underestimation nonequilibrium solvation from linear response theory compensating overestimation of excitation energies from the DFT methods we employ. Results here show less accurate results for the TC-3-NKG example if BHandLYP is used. State specific nonequilibrium solvation applied to TC-3 fluorescence with ethanol the solvent gave similar results to equilibrium solvation indicating nonequilibrium solvation effects are small for fluorescence in this case.

Part of the error in comparing computed *E*_v,a_ to experimental *E*_abs_ = *hc*/*λ*_max_ is the implicit assumption that electronic excitations occur from an exactly specified molecular geometry. The error can be avoided if *λ*_max_ from a computed vibronic spectrum is used however, there can be difficulties with this comparison because electronic structure methods that predict accurate band shape due to vibronic transitions may not also predict accurate position in the spectrum. Bloino *et al.* (2015) found that functionals that give accurate position in the spectrum vastly underestimate the intensity of the major peak indicated by experiment for four coumarin dyes but give accurate results for the low frequency peak with maximum near *E*_00_.^7^ The situation is simpler for cyanine dyes for which this low frequency band is also the most intense one in experimental spectra and one can predict *λ*_max_ for the spectrum based on this band that is also described well by functionals that give accurate position in the spectrum. Our finding that AH FC analysis and the QD method give more accurate *λ*_max_ when B3LYP is used rather than BHandHLYP may be due to the fact that for cyanine dyes, the low frequency peak in the vibronic spectrum has the highest intensity. The QD method if applied to coumarin dyes would be appropriate for predicting *λ*_max_ for this lowest frequency band but not the band corresponding to the most intense peak in the experimental spectrum.

Alternatively, following the example of Bloino *et al.* (2015), one might use two basis sets, for example, ωB97x for accurate overall band shape shifted by PBE0 results for accurate *λ*_max_. Translation of this approach to the QD model would mean using *E*_v,a/f_ and *E*_ad_ from ωB97x to compute *E*_QD,a/f_ and then shifting their position in the spectrum by Δ*λ* = *λ*_VE,PBE0_ − *λ*_VE,XCF_ where XCF, the functional used for more accurate vibronic structure, might be ωB97x. Using *E*_v,a/f_ and *E*_ad_ from ωB97x would implicitly include effects of contributions from the vibrations contributing to the higher frequency peak more accurately described by ωB97x than by PBE0 which gives more accurate position. This approach may give better accuracy in comparison to a vibronic spectrum for which symmetric Gaussian broadening due to solvent does not allow these peaks to be distinguished.

**Fig. 9:**
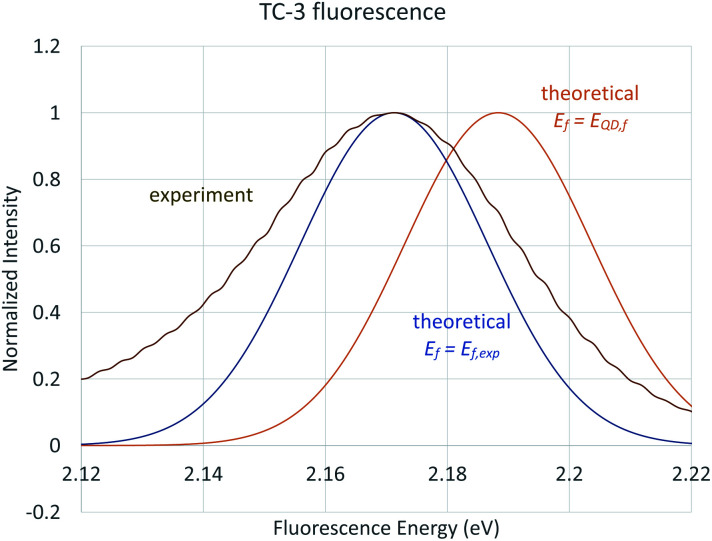
Experimental fluorescence (brown), theoretical Gaussian broadening centered at the experimental fluorescence energy (blue), theoretical Gaussian broadening centered at *E*_QD,f_ (orange).

The reliance of the QD method on only *E*_v,a/f_, *E*_ad_, and *E*_00_ precludes its description of temperature dependent effects that cause both broadening of spectral lines and shifting in the spectrum. The TD-FC spectra we present show broadening at 298 K compared to 0 K but do not include temperature dependent solvent effects. Santoro *et al.* (2015) present a simple classically based approach to estimate solvent broadening based on a symmetric Gaussian distribution of solvent energies. The standard deviation in solvent energies is estimated according to temperature and difference in vertical transitions with nonequilibrium and equilibrium implicit solvation, *σ*^2^ = 2*k*_B_*T*(*E*^neq^_v_ − *E*^eq^_v_).^83^ The results of state specific nonequilibrium solvation for TC-3 plugged into this equation give *σ* = 0.15 eV from B3LYP and *σ* = 0.14 eV from M06HF. The Gaussian peak from the B3LYP *σ* is shown in Fig. 9 compared to the experimental fluorescence spectrum of TC-3.

In addition to solvent broadening with increased temperature, temperature dependent solvent effects may cause shifts in *λ*_max_. Huppert *et al.* (2011) show the spectrum of curcumin in ethanol at 84 K to include three peaks similar to the coumarin dyes, the central peak being the most intense and having *λ*_max_ at 2.44 eV (509 nm) in excellent agreement with *E*_v,f_ = 2.47 eV (502 nm) from M06. They report the 0–0 peak at *E*_v,f_ = 2.59 eV (478 nm) while QD model gives QD_v,f_ = 2.50 eV (496 nm) and an error of ≈ 0.1 eV much like the cyanine dye examples. Huppert *et al.* report the peaks in the curcumin vibronic emissions spectrum are separated by 1270 cm^−1^, 0.157 eV. Assuming the same band structure for the vibronic absorption spectrum at 84 K and *λ*_max_ as reflecting the central peak, allows an estimate of the 0–0 peak in the absorption spectrum to be at 2.76 eV and an error in *E*_QD,a_ of − 0.15 eV. Given that QD method applies to the low frequency peak, these errors are similar to those for the cyanine dyes.

Huppert *et al.* (2011) explain the dramatic redshift to 2.20 eV (563 nm) with increase in temperature to 298 K according to solvent dynamics that disrupt the intramolecular H-bond in the curcumin keto–enol system causing nonradiative decay by allowing the ring systems on either end to twist with respect to each other.^82^ They report the position and shape of the spectrum are nearly temperature independent at temperatures less than 165 K at which solvent dynamics have time scales similar to the excited state lifetime.

Temperature dependent solvation effects are more of a concern for curcumin and coumarin dyes than for cyanine dyes because curcumin and cyanine dyes have fluorescent life times in ethanol on order of nanoseconds, two order of magnitude greater than for cyanine dyes in ethanol.^82,84,85^ Temperature dependent solvent effects for cyanine dyes therefore would require much higher temperature.

Excited state twisting of molecules consisting of aromatic groups bridged by a conjugated carbon chain is a mechanism of nonradiative decay leading to lower fluorescence intensity. Prevention of such twisting at low temperatures or in an environment where the dye has more restricted movement enhances fluorescence and this property can be used in biosensors. This motivated computations with TC-1 in a DNA minor grove model to see if this environment would restrict the twisting found in the TC-1 excited state geometry in PCM water. EG has an enhanced and redshifted fluorescence in the DNA minor groove EG-M was also the subject of calculations in our DNA minor groove model.

### ONIOM TC-1 and EG-M in the DNA minor groove model

**Fig. 10:**
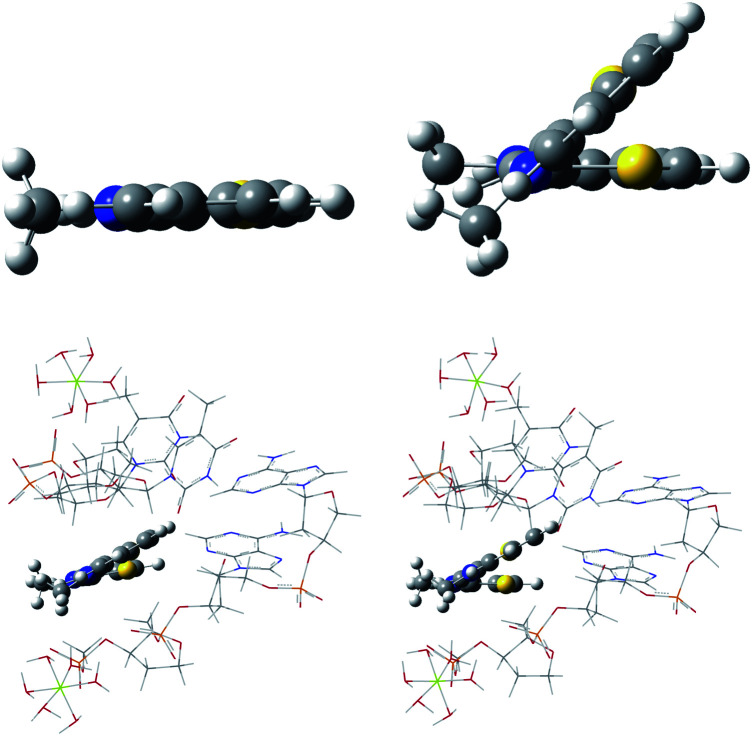
Optimized geometries of TC-1: ground state in water (top left), excited state in water (top right), ground state in the DNA minor groove (bottom left), excited state in the DNA minor groove (bottom left).

The TC-1 cation in its ground state was predicted to have a twist along the methine chain when complexed in the DNA minor groove according to the ONIOM model used here. This is in contrast to its planar geometry predicted in water. See Fig. 10. The excited state, while optimizing with B3LYP and water as the PCM solvent drastically twists with puckering similar to the ethylene first excited state.^75^ A minimum energy geometry was not found. The first excited state of ethylene is an example used to test DFT methods designed for use in cases that are multireference in the context of wavefunction methods.^75^ Excited state geometries were successfully optimized for TC-1 in PCM water and in the ONIOM DNA minor groove model using ONIOM MN15:UFF. See Fig. 10. The change in excited state twisting is less for TC-1 in the DNA minor groove (change in dihedral S_1_C_2_C_6_S_2_ 17° → 28° GS → ES) than in water (change in dihedral S_1_C_2_C_6_S_2_ 0° → 43° GS → ES) indicating a more restricted geometry in the DNA minor groove setting consistent with enhanced fluorescence displayed by TC-1 in a biological setting. Attempts at calculating a vibronic spectrum for TC-1 in the DNA minor groove and in water were not successful. Large expected errors in the ZPE due to a large number of normal modes in the DNA model precluded use of the QD method. EG-M showed one imaginary vibrational frequency in its excited state in the ONIOM DNA minor groove calculations precluding further analysis by FC or QD methods. The QD model results for EG-M still allow some interpretation as to the nature of the redshift of EG when complexed to λDNA though our DNA model does not contribute to this interpretation.

### Possible explanation of redshift in EvaGreen absorbance in presence of λDNA

The fused ring structure of EG-M is different than the THIA and INDO cyanine dyes and the underestimation by *E*_QD,a_ of EG-M experimental *E*_abs_ for free EG seems to imply higher frequency vibronic transitions contribute to the major band for this molecule similar to the curcumin and coumarin examples. If this is the case, the dominant low frequency band that emerges when EG is complexed to λDNA with *E*_abs_ = *hc*/*λ*_max_ = 2.50 eV may be due to a low frequency band well described by *E*_QD,a_ = 2.53 eV.

## Conclusions

Vertical transition energies, *E*_v,a/f_ from the equilibrium geometry of the initial electronic state in a transition are frequently considered to be representative of the *E*_abs/fl_ = *hc*/*λ*_max_ from experiment. It is shown here *E*_v,a/f_ for THIA and INDO cyanine dyes occur in the Franck–Condon region predicted by TDDFT and FC analysis but correspond to vibrations far from the most intense peak and to vibrational structure that is mostly washed out in experimental spectra in solution. Low wavenumber vibrations contribute to the major peak in the computed vibronic spectra with *E*_abs/fl_ = *hc*/*λ*_max_ showing a 10-fold improvement in accuracy compared to *E*_v,a_. The computed vibronic spectra are based on the quantum version of the FC principle which relies neither on exactly specified molecular geometries before, during, or after a transition nor specified duration for an electronic transition in comparison to the period of molecular vibrations involved in it.

A classically inspired approach is developed which corresponds to vertical transitions from an averaged molecular geometry appropriate in a way analogous to the r-centroid that preserves kinetic energy before and after an electronic transition in accord with the classical version of the FC principle. We refer to this classically inspired approach as the quarter difference (QD) method based on the way it utilizes the harmonic approximation. Application of the quantum FC and QD methods give better accuracy than *E*_v,a/f_ showing a 5-fold improvement in accuracy compared experiment.

The QD model is an implementation of an adiabatic approximation appropriate for low frequency vibronic transitions near the 0–0 transition as appropriate for a classical approximation. This is the major peak in spectra of cyanine dyes so the approach is particularly well suited for them. Extension of the QD model to non-cyanine dyes was tested using M06 computations which gave accurate position in the spectrum for curcumin, but with inaccurate overall band shape due to underestimation of the intensity of higher frequency bands as seen in the literature for coumarin dyes. Though the vibronic spectrum of curcumin in ethanol was not obtained, the QD model applied to the low frequency band and compared to the low frequency band from experiment gave similar accuracy as for cyanine dyes. Dyes with long lifetimes compared to solvent dynamics such as curcumin and coumarin dyes are more prone to temperature dependent solvation effects on their spectra and comparisons to results from the QD model is more complicated but not impossible as was demonstrated with the curcumin example.

Computed vibronic spectra at 298 K show reduced intensity at this temperature compared to 0 K suggestive of experimental spectra where solvent viscosity, aggregation, or complexation to biomolecules restrict motion of the dye. A 2-layer ONIOM model of the DNA model shows the geometry of the TC-1 dye excited state has a restricted twist in its geometry compared to that in water and perhaps conducive to enhance fluorescence in this setting. *E*_QD,a_ for EG-M matches the longer wavelength peak that becomes dominant when EG is bound to λDNA. This could indicate a change in EvaGreen vibrations due to this complexation perhaps damping those responsible for the higher frequency peak in free EG and enhancing low frequency vibronic transitions well described by the QD model.

## Conflicts of interest

There are no conflicts to declare.

## Supplementary Material
